# New compound heterozygous variants of the cholinergic receptor nicotinic delta subunit gene in a Chinese male with congenital myasthenic syndrome: A case report

**DOI:** 10.1097/MD.0000000000008981

**Published:** 2017-12-22

**Authors:** Huiru Feng, Hongyu Zhou

**Affiliations:** Department of Neurology, West China Hospital, Sichuan University, Chengdu, China.

**Keywords:** c.423G>C (exon5), c.59G>A (exon2), cholinergic receptor nicotinic delta subunit (CHRND), congenital myasthenic syndrome (CMS)

## Abstract

**Introduction:**

Congenital myasthenic syndromes (CMS) are a group of genetic disorders that stem mostly from molecular defects in nicotinic acetylcholine receptors (AChRs). Defects in the cholinergic receptor nicotinic delta subunit (*CHRND*) gene can cause a series of myasthenic syndromes. Here, we report 2 new compound heterozygous variants of the *CHRND* gene in a Chinese male with CMS.

**Case presentation:**

A 43-year-old Chinese male presented with progressive muscle weakness, difficulty chewing, and an inability to lift his head from the time he was 8 years old. He was treated with pyridostigmine, which was partially effective. Two weeks prior, he was hospitalized for dyspnea. Upon examination, he was unable to drum his cheeks and exhibited fatigable muscle weakness and facial muscle atrophy. Sequencing of his exome revealed 2 previously unreported mutations in CHRND, c.59G>A (exon2) and c.423G>C (exon5).

**Conclusions:**

We identified a new mutational site that contributes to the onset of CMS.

## 1 Introduction

Congenital myasthenic syndromes (CMS) are a rare heterogeneous group of inherited neuromuscular disorders that are caused mainly by mutations in genes coding for proteins that are expressed at the neuromuscular junction. Since the 1970s, CMS have been classified according to the location of the mutant protein, such as presynaptic, synaptic basal lamina-associated, defects in the acetylcholine receptor (AChR), defects in endplate development and maintenance, congenital defect of glycosylation, and other myasthenic syndromes. The most common cause of CMS is defects in the AChR.^[1]^

The AChR in the muscle can be classified as one of the following 5 different types: 2 alpha (CHRNA1) subunits and 1 each of the beta (CHRNB), gamma (CHRNG) and delta (CHRND), and epsilon (CHRNE) subunits. An integral membrane protein has a subunit composition of α2βδε at the adult endplate or α2βδγ at the fetal endplate and extrajunctional regions. The genes encoding the α (*CHRNA1*), δ (*CHRND*), and γ (*CHRNG*) subunits are located at different loci on chromosome 2q, and those encoding the β (*CHRNB*) and ε (*CHRNE*) subunits are located at different loci on chromosome 17p.^[2]^

Mutations in the *CHRND* gene, which encodes the δ subunit of an integral membrane protein, can cause 4 main types of CMS: multiple pterygium syndrome lethal (MUPSL), slow-channel CMS (SCCMS), fast-channel CMS (FCCMS), and AChR deficiency CMS.^[2]^ Several transcript variants encoding different isoforms have also been found for this gene,^[2]^ and different types of CMS have different clinical manifestations and pathophysiologies. Multiple pterygium syndrome is a condition that is evident before birth with webbing of the skin (pterygium) at the joints and lack of muscle movement (akinesia). Lethal multiple pterygium syndrome is fatal before birth or very soon after birth.^[3]^ AChR deficiency CMS can result from recessive missense, nonsense, or splice site and promoter region mutations in the *CHEND* gene, causing AChR deficiency, and patients with this type respond well to pyridostigmine.^[4]^ Slow-channel syndrome is caused by dominant mutations in ligand-binding or pore domains of the AChR that result in prolonged synaptic currents and action potentials, and patients with slow-channel mutations in their AChRs deteriorate on pyridostigmine.^[5]^ Fast-channel syndrome is caused by a recessive mutation in 1 AChR subunit allele, accompanied by a null or low-expressing mutation or, rarely, by another fast-channel mutation on the other allele. Fast-channel syndrome is physiologically opposite of the slow-channel syndrome in that the endplate currents decay abnormally fast, the channel openings are abnormally brief, and the amplitudes of synaptic currents and potentials are reduced, owing to a decreased probability that the AChR channel is opened by physiological concentrations of acetylcholine. Patients with this syndrome respond well to pyridostigmine.^[6]^ Our case report involves a male patient with CMS, and 2 novel mutant variants of CHRND were found to be the likely causative agents.

## 2 Case report

### 2.1 Clinical findings

A 43-year-old Chinese male presented with progressive muscle weakness, difficulty chewing, and an inability to lift his head since he was 8 years old. He achieved remission of symptoms after treatment with pyridostigmine, but the remission was incomplete. Over a year ago, the abovementioned symptoms worsened. He increased the dosage of pyridostigmine to 60 mg 5 times a day. However, the symptoms were not evidently relieved. Twenty-six days prior, he accidently fell and broke his left leg. Then, he was hospitalized and had a pubis fracture operation performed. General anesthesia was used during the fracture operation. After the surgery, he had difficulty breathing (shortness of breath and flustered), which was accompanied by sinus tachycardia. Then, he was transferred to the respiratory intensive care unit. After the anti-infection and symptomatic treatment, the trachea cannula was removed, and the mechanical ventilator assistance was ended. However, this was accompanied by dyspnea and tachycardia with activities. His birth and past medical histories were otherwise unremarkable. There is no parental consanguinity or family history of muscle disorders.

Upon examination, he had a body mass index (BMI) of 20.3 kg/m^2^, was unable to drum the cheeks, and had fatigable muscle weakness [proximal upper extremity Medical Research Council (MRC) grade 4/5; distal upper extremity MRC grade 4+/5; proximal lower extremity MRC grade 4+/5; distal lower extremity MRC grade 4/5; and dorsal and plantar flexors MRC grade 5/5]. His facial muscles were atrophied, but the bilateral gastrocnemius muscles were slightly hypertrophied. This was accompanied by femoral hyperreflexia. Other than the spinal scoliosis, the remainder of the examination was normal.

**Figure 1:**
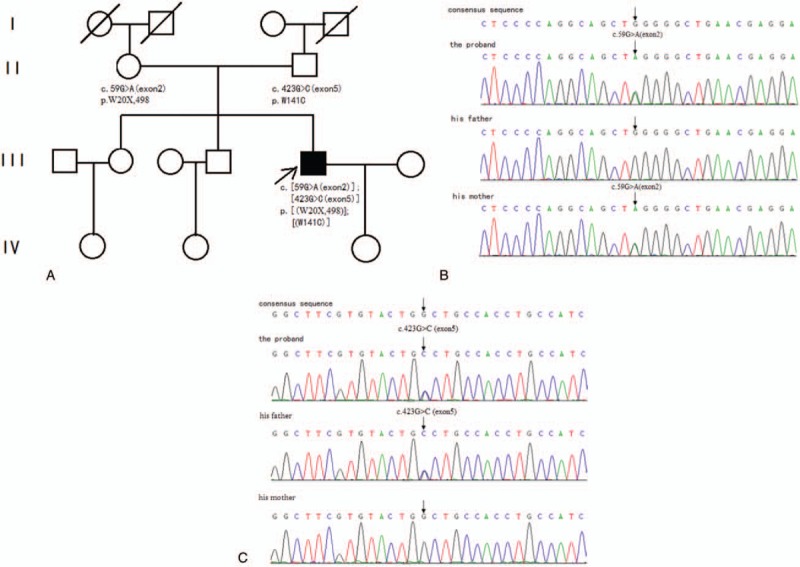
(A) Pedigree of the family in which the compound heterozygous mutations in CHRND were found. The arrowhead indicates the proband. Black-filled symbols represent the subjects with FCCMS. White symbols represent the unaffected family members. The variants found are represented below with each individual showing segregation of the compound heterozygous mutation of the disease. The diagonal lines indicate dead individuals. (B) Sequencing traces from a region within exon 2 of the *CHRND* gene from a healthy individual, the patient, and his parents. The 5-nucleotide changes c.59G>A (exon2) are indicated. (C) Sequencing traces from a region within exon 5 of the *CHRND* gene from a healthy individual, the patient, and his parents. The 5-nucleotide changes c.423G>C (exon5) are indicated. However, his brother and sister, their children, and the proband's grandmother have not had genetic tests performed.

The hematological and biochemical investigations, including a full blood count, inflammatory markers, glucose, and renal and liver function tests, were normal. The immunological tests, that is, anti-neutrophil cytoplasmic antibodies (ANCA), coagulation profile, thyroid functions, serum lactate, and creatine kinase level, were normal. The anti-AChR and skeletal muscle receptor tyrosine kinase (MUSK) antibodies were negative in the serum. No obvious abnormality was observed in the heart, abdomen, and urinary tract ultrasound. Thoracolumbar scoliosis was observed on chest computed tomography (CT) scans. Repetitive nerve stimulation at low rates showed a marked decrement (>30%) in the accessory nerve (normal at a high rate). His electromyography showed a myogenic lesion in the right dorsal interosseous muscle. Exome sequencing showed 2 novel variant mutations [c.59G>A (exon2) and c.423G>C (exon5), Fig. 1A–C], which were compound heterozygous variants, on the *CHRND* gene according to a phenotype–genotype correlation. The 2 mutations have never been reported according to the OMIN, HGMD, and CLINVAR databases. No occurrence of mtDNA mutations or copy number variations (CNVs) were found. He remained functionally independent with the pyridostigmine treatment.

### 2.2 Materials and methods

Venous blood samples were obtained from the CMS patient and his unaffected parents. All studies were performed with informed consent from the patient's parents and were approved by the institutional ethics review board. Genomic DNA was isolated using BloodGen Midi Kit (CWBIO, China) according to the manufacturer's recommendations.

#### 2.2.1 Next-generation sequencing (NGS) and DNA sequence analysis

The genomic DNA sample was sheared by sonication. The sheared genomic DNA was then hybridized using the NimbleGen 2.0 probe sequence capture array by Roche to enrich the exonic DNA (Joy Orient, China). The libraries were first tested for enrichment by qPCR (polymerase chain reaction) and for size distribution and concentration using the Agilent Bioanalyzer 2100. The samples were then sequenced on an Illumina Hiseq2500 platform (Illumina, Hayward CA). Two parallel reactions were performed for each sample.

#### 2.2.2 Data filtering, mapping, and variant detection

The exon-enriched DNA was sequenced using the Illumina Hiseq2500 platform according to the manufacturer's instructions (Illumina). The raw image files were processed using BclToFastq (Illumina) for base calling and generating raw data. The low-quality variations were filtered using a quality score ≥20 (Q20). The sequencing reads were aligned to the NCBI human reference genome (hg19) using BWA. SAMtools and Pindel were used to analyze the single nucleotide polymorphisms (SNPs) and indels in the sequence.

#### 2.2.3 Data analysis

The data analysis was performed as follows:

1. Synonymous changes and SNPs with a minor allele frequency (MAF) greater than 5% were removed.
2. Nonsynonymous changes were filtered using SIFT software.
3. The function of the mutated genes and their relationship to the disease were analyzed.

#### 2.2.4 Mutation confirmation using Sanger sequencing

Sanger sequencing was used to confirm the mutation in the proband. The PCR primers and lengths of the PCR products are summarized in Table 1.

**Table 1:**
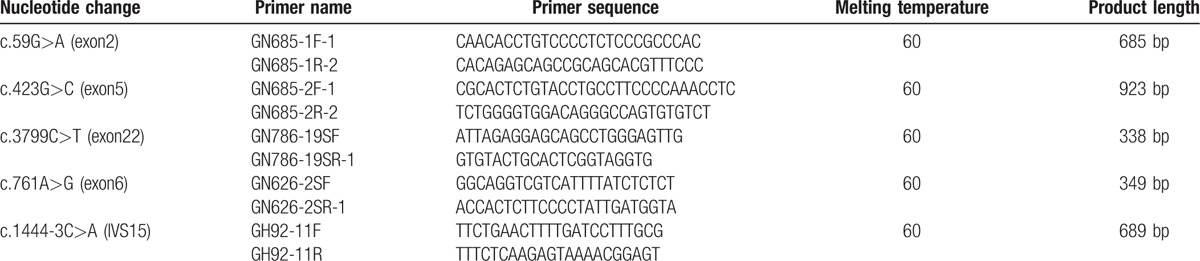
Primers and product lengths of genes.

The PCR products were sequenced on an ABI 3730XL system, analyzed using DNASTAR software, and compared using an mRNA template (ATP8B1: NM_005603, IGSF1: NM_001170961.1).

Finally, in addition to certain mutations with a low probability, 4 mutations were found that may have caused myasthenia in the patient (Table 2). On the basis of a phenotype–genotype correlation, the 2 mutations [c.59G>A (exon2) and c.423G>C (exon5) mutations] in the *CHRND* gene, which may cause FCCMS, were most likely pathogenic mutations.

**Table 2:**
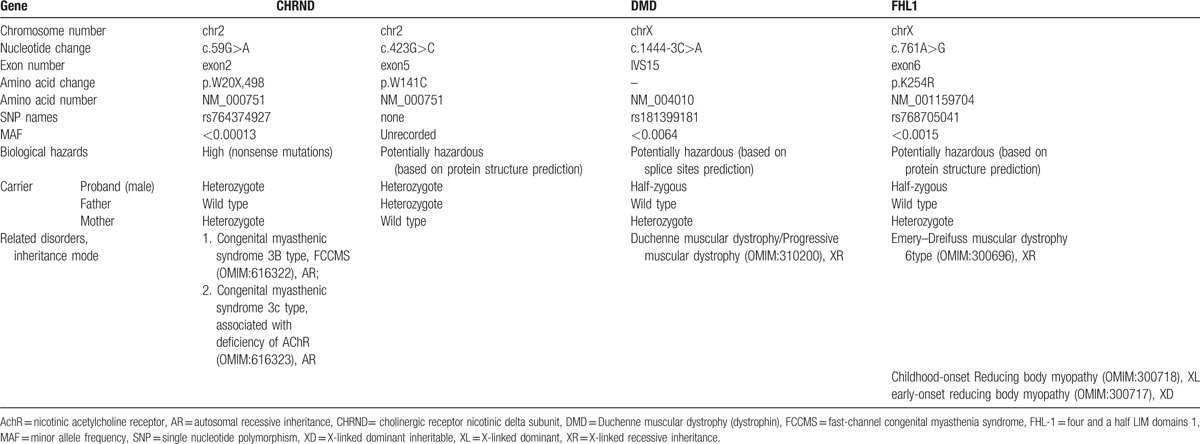
Genetic mutations with a high pathogenicity.

No occurrences of mtDNA mutations or CNVs were observed in the mitochondrial DNA sequencing and CNS analyses.

## 3 Discussion

On the basis of the phenotype–genotype correlation (Table 2), the mutations in the *CHRND* gene were pathogenic. Our patient has a relatively mild condition and responds well to pyridostigmine. He was diagnosed with 2 new mutations [c.59G>A (exon2) and c.423G>C (exon5)] in the *CHRND* gene. The first mutation is predicted to terminate protein translation (p.W20X, 498), resulting in the truncation of the delta subunit in its extracellular domain. This variant is SNP rs764374927. According to the sequencing data presented here, this duplication is predicted to be heterozygous and inherited from his mother, who carried the c.59G>A (exon2) heterozygous variant. The second mutation is predicted to convert the Trp codon to Cys at position 141 of the protein (p.W141C). According to the sequencing data presented here, this duplication is predicted to be heterozygous and inherited from his father, who carried the c.59G>A (exon2) variant. Thus, the patient carries the following compound heterozygous mutations: c.59G>A (exon2) and c.423G>C (exon5); p.W20X,498; and p.W141C. The new compound heterozygous variants of the nicotinic AChR gene (*CHRND*) were likely the cause of CMS. The two variants have not been previously documented in large genetic databases. On the basis of the phenotype–genotype correlation, the MUPSL and SCCMS types of CMS could be excluded.^[2]^ However, we could not conclude whether the patient was afflicted with the FCCMS or AChR deficiency type based on clinical manifestations and whole exome sequencing, owing to the diversity of functional defects caused by mutations in AChR subunits. Additional studies on channel burst durations would provide a precise diagnosis.^[7]^

Finally, the patient was diagnosed with CMS (most likely the FCCMS or AChR deficiency type). However, the genetic diagnosis was confirmed 26 years after the onset only when the respiratory muscles became involved, and the previous diagnosis of progressive muscular dystrophy was revised.

In conclusion, our aim is to highlight the importance of molecular diagnosis in CMS and report novel compound heterozygous variants of the nAChR gene (*CHRND*) that can cause CMS [c.59G>A (exon2) and c.423G>C (exon5) mutations]. In most cases of CMS, clinical, EMG, and molecular genetic studies point to the correct diagnosis. Therefore, for patients with myasthenic syndrome, early genetic testing will help neurologists make a definitive diagnosis and prevent a misdiagnosis.^[8]^ When possible, in vitro microelectrode or single-channel patch-clamp recordings are required to define the pathomechanism and appropriate therapy.^[9]^ Regarding treatment, the drugs that benefit 1 type of CMS can be ineffective or harmful in another type.^[6,10,11]^ For example, patients harboring low expression or fast-channel mutations in AChRs show improvement with cholinergic agonists, whereas the condition in patients with slow-channel mutations in AChRs deteriorates with these drugs.^[6]^ Therefore, a molecular diagnosis is essential to inform the choice of therapy and predict the onset rate in offspring. Although CMS is of low incidence, a molecular diagnosis cannot be ignored in patients with myasthenic syndrome.
